# Exploring the Role of Mobile Apps for Insomnia in Depression: Systematic Review

**DOI:** 10.2196/51110

**Published:** 2024-10-18

**Authors:** Yi-Hang Chiu, Yen-Fen Lee, Huang-Li Lin, Li-Chen Cheng

**Affiliations:** 1 Department of Psychiatry Wan Fang Hospital Taipei Medical University Taipei City Taiwan; 2 Psychiatric Research Center Wan Fang Hospital Taipei Medical University Taipei City Taiwan; 3 Department of Information and Finance Management National Taipei University of Technology Taipei City Taiwan; 4 Department of Psychiatry Chang Gung Memorial Hospital, Linkou Taoyuan Taiwan; 5 College of Medicine Chang Gung University Taoyuan Taiwan

**Keywords:** depression, insomnia, chatbots, conversational agents, medical apps, systematic review, technical aspects, PRISMA

## Abstract

**Background:**

The COVID-19 pandemic has profoundly affected mental health, leading to an increased prevalence of depression and insomnia. Currently, artificial intelligence (AI) and deep learning have thoroughly transformed health care–related mobile apps, offered more effective mental health support, and alleviated the psychological stress that may have emerged during the pandemic. Early reviews outlined the use of mobile apps for dealing with depression and insomnia separately. However, there is now an urgent need for a systematic evaluation of mobile apps that address both depression and insomnia to reveal new applications and research gaps.

**Objective:**

This study aims to systematically review and evaluate mobile apps targeting depression and insomnia, highlighting their features, effectiveness, and gaps in the current research.

**Methods:**

We systematically searched PubMed, Scopus, and Web of Science for peer-reviewed journal articles published between 2017 and 2023. The inclusion criteria were studies that (1) focused on mobile apps addressing both depression and insomnia, (2) involved young people or adult participants, and (3) provided data on treatment efficacy. Data extraction was independently conducted by 2 reviewers. Title and abstract screening, as well as full-text screening, were completed in duplicate. Data were extracted by a single reviewer and verified by a second reviewer, and risk of bias assessments were completed accordingly.

**Results:**

Of the initial 383 studies we found, 365 were excluded after title, abstract screening, and removal of duplicates. Eventually, 18 full-text articles met our criteria and underwent full-text screening. The analysis revealed that mobile apps related to depression and insomnia were primarily utilized for early detection, assessment, and screening (n=5 studies); counseling and psychological support (n=3 studies); and cognitive behavioral therapy (CBT; n=10 studies). Among the 10 studies related to depression, our findings showed that chatbots demonstrated significant advantages in improving depression symptoms, a promising development in the field. Additionally, 2 studies evaluated the effectiveness of mobile apps as alternative interventions for depression and sleep, further expanding the potential applications of this technology.

**Conclusions:**

The integration of AI and deep learning into mobile apps, particularly chatbots, is a promising avenue for personalized mental health support. Through innovative features, such as early detection, assessment, counseling, and CBT, these apps significantly contribute toward improving sleep quality and addressing depression. The reviewed chatbots leveraged advanced technologies, including natural language processing, machine learning, and generative dialog, to provide intelligent and autonomous interactions. Compared with traditional face-to-face therapies, their feasibility, acceptability, and potential efficacy highlight their user-friendly, cost-effective, and accessible nature with the aim of enhancing sleep and mental health outcomes.

## Introduction

### Background

Insomnia and depression have been significantly correlated with one another. Clinically, the incidence of insomnia is higher in patients with depression than in those without, and the symptoms tend to be more severe [1]. In addition to the fact that depression is often accompanied by symptoms of insomnia, insomnia itself is a risk factor for depression [2,3], and severe insomnia can exacerbate depressive symptoms. Insomnia may also serve as a prodromal symptom of depression, as many patients with depression also report insomnia complaints. According to Gebara et al [4], treating insomnia in patients with both depression and insomnia symptoms can contribute to emotional improvement. The treatments for depression and insomnia are interconnected; improvements in depression often lead to relief from insomnia. However, specifically addressing insomnia can also facilitate the alleviation of depressive symptoms.

In clinical practice, the treatment of insomnia primarily involves cognitive behavioral therapy (CBT) and medication, with most patients responding well to these approaches. Particularly during the COVID-19 pandemic, when patients were unable to visit medical institutions for in-person treatment, telemedicine and mobile device–based medical services became increasingly important [5]. As in-person hospital visits become less feasible, the significance of telemedicine models has increased. While these models have not entirely replaced traditional medical practices, they have proven effective in assisting with the treatment of various conditions, including psychiatric disorders. Examples of such models include internet-based services, mobile devices, and software apps [6-8]. Telemedicine models, including video clinics and technology-assisted mobile health (mHealth) approaches, as well as flexible eHealth solutions, have become pivotal in addressing the escalating demand for mental health support amid the scarcity of conventional health care resources [9]. These approaches aim to tackle the challenges posed by global mental health issues and shortages in traditional health care facilities [10,11].

Recently, artificial intelligence (AI) apps have significantly advanced psychiatric diagnostic assistance, symptom tracking, course prediction, morbidity risk assessment, and mental health education [12]. These apps are increasingly being utilized as therapeutic aids, offering advantages such as high accessibility, convenience, low cost, and sustainability. Notably, innovative AI-assisted therapies, such as chatbots, can provide ongoing follow-up and extended treatment while mitigating the stigma associated with disclosing mental illness, thereby enhancing personal well-being [13-17].

Considering past research, to the best of our knowledge, no review papers have examined mobile apps targeting insomnia in individuals with depression. Therefore, this systematic review aims to comprehensively categorize the thematic features of mobile apps designed for addressing insomnia and depression, shedding light on strategies to improve insomnia among individuals with depression. This study offers a systematic overview of the research field, enhances understanding, and reveals new potential applications and research directions.

### Related Work

To gain a deeper understanding of the impact of technological advancements on mobile apps in psychiatry, we organized and evaluated systematic and literature reviews published in the past 5 years. This 5-year limit for the literature search is justified by the rapid advancements in AI and mHealth technology. In recent years, significant advancements in medical technologies have rendered older studies potentially less relevant in the current context [18-21]. This study aimed to capture the evolving trends in mental health–related mobile apps resulting from these technological advancements, providing comprehensive insight into this field.

We assessed recent systematic and literature reviews, revealing a focus on 2 main aspects: insomnia and depression. Regarding insomnia, 8 papers [22-29] primarily addressed the application of CBT principles [22], smartphone app support for sleep self-management [23,24], the usability and principles of smartphone apps for insomnia [25,27], wearable device–based sleep monitoring [26], and the study of CBT within electronic mental health apps [28,29]. Regarding depression, 9 papers [30-38] examined topics such as therapist-supported internet-based CBT [30,31,37], user experience analysis of depression apps [32,34], the adoption of mobile apps in the domains of depression and anxiety [33,35,36], and a review of mobile mental health apps [38].

However, there is a lack of comprehensive research on patients with depression who also experience insomnia symptoms. Although depression and insomnia are common comorbidities in clinical settings, the exploration of these specific conditions has not been thoroughly investigated. Therefore, this study primarily aimed to fill this research gap and provide a more comprehensive understanding of the development and application of mobile apps for patients with both depression and insomnia symptoms.

## Methods

### Study Identification and Selection

This study adhered to the PRISMA (Preferred Reporting Items for Systematic Reviews and Meta-Analyses) guidelines [39] for the literature search and article selection (Multimedia Appendix 1). Details regarding methodological protocol amendments and clarifications can be found in Multimedia Appendix 2.

### Search Strategy

In the first step, a comprehensive systematic search was conducted across multiple databases, including PubMed, Scopus, and the ISI Web of Science, using keywords such as “mobile applications,” “chatbots,” “insomnia,” “insomnia treatment,” and “depression.” The complete list of search terms is available in Multimedia Appendix 3. The search criteria were further refined by specifying additional constraints, including publication in peer-reviewed academic journals in English and restricting the time frame to articles published between 2017 and early 2023. This ensured that the selected literature from the most recent 5 years aligned with current technologies and the latest research findings. Systematic reviews typically limit inclusion to studies published within a specified time frame to ensure relevance and capture current evidence. This approach helps maintain the validity of the findings by focusing on recent developments and excluding outdated or less pertinent research [18-21]. A preliminary search yielded 383 articles.

### Screening Process

Two experienced researchers meticulously screened the studies retrieved from the databases to ensure that they (1) focused on mobile apps addressing both depression and insomnia, (2) involved young or adult participants, and (3) provided data on treatment efficacy. The screening process was conducted in 2 stages: first, they independently screened the titles and abstracts to identify studies that potentially met the inclusion criteria; second, they reviewed the full texts of the selected studies to determine final inclusion. During the coding process, the researchers read the papers and engaged in discussions to reach a consensus on any inconsistencies in the codes. The risk of bias was assessed using the Cochrane Risk of Bias framework [40].

The screening process was conducted based on various aspects, including research topic characteristics, participant demographics, app features, therapeutic efficacy, operating platforms, technical foundations, feasibility, and acceptability. This approach ensured that the selected studies aligned with the research objectives, thereby enhancing the quality and relevance of the final sample. After reviewing the titles, abstracts, and full texts, 18 articles were included in the final sample. Exclusion criteria encompassed nonacademic journal articles, lack of relevance to the topic, insufficient discussion content, and methodological limitations. This review process ensured the high quality and relevance of the final sample to the research objectives.

### Data Extraction and Quality Assessment

#### Data Extraction

A preliminary coding scheme was developed based on an initial review of the included articles. Subsequently, 2 researchers (YHC and HLL), utilized this scheme to code 10 articles, after which they convened to discuss the practicality of the initial and potential additional coding categories. Throughout the coding process, the 2 experienced researchers read the papers and engaged in discussions to reach a consensus on any inconsistencies in the coding. The final coding scheme comprised 7 dimensions: Research topics (eg, cognitive and emotional support, cognitive behavioral interventions, psychological education, sleep diaries, self-help psychotherapy, conversational agents, examining depression symptoms, and digital behavioral interventions); Participants (eg, young people or adults); App functions (eg, early detection, assessment and screening, counseling and mental support, and CBT); Therapeutic efficacy (eg, the effectiveness of mobile apps as sleep intervention tools, the effectiveness of chatbots in improving sleep quality and insomnia symptoms, and the effectiveness of chatbots in improving depression and its symptoms); Operating platforms (eg, Android, iOS, Android and iOS, multiplatform support, and unreported operating platforms); Technical foundation and feature characteristics (eg, AI chatbots, the ExpiWell app, RASA-based open-source conversational AI); and Feasibility and acceptability (ie, the acceptability and feasibility of chatbots, mobile apps as sleep intervention tools, and mobile apps providing depression intervention and emotional support).

#### Quality Assessment

The risk of bias was assessed using the Cochrane risk of bias framework [40]. One reviewer (YHC) initially evaluated each study’s risk of bias, which was then verified by a second reviewer (HLL). The assessment encompassed sequence generation, allocation concealment, blinding, completeness of outcome data, selective reporting, and other potential biases. Any discrepancies were resolved through consensus between the 2 reviewers.

## Results

### Study Inclusion

**Figure 1:**
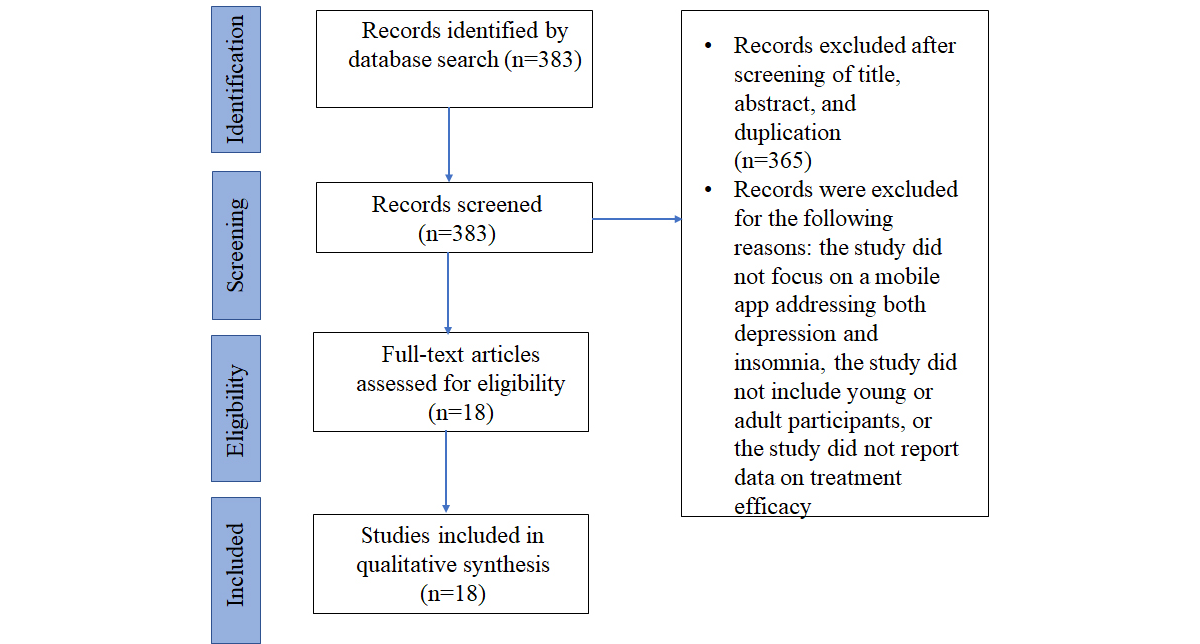
PRISMA (Preferred Reporting Items for Systematic Reviews and Meta-Analyses) flowchart illustrating the literature search and selection process of studies included in this review.

Of the 383 abstracts initially identified, 365 were excluded based on title and abstract screening, as well as duplication; 18 full-text articles met the inclusion criteria. The search process is illustrated using the PRISMA flowchart (Figure 1).

### Study Characteristics

Table 1 presents the 18 identified apps, categorizing their primary functions in addressing insomnia and depression. This categorization enhances the understanding of the correlation between insomnia and depression, offering a comprehensive perspective [23,24]. Chatbots and smartphone apps are commonly classified as mobile apps. We explored the shared factors and therapeutic approaches within these mobile apps that contribute to improving sleep and mental health.

**Table 1:** Study characteristics regarding insomnia and depression chatbots in the review (N=18).

Design and app			Study		Aim		Total sample (age)
**Cognitive behavioral interventions (n=10)**							
	Woebot	[5]		Assessed the feasibility of implementing an app-based cognitive behavioral therapy intervention for adolescents undergoing treatment for depression. This investigation focused on the intervention’s acceptability, practicality, and safety.		N=18 (13-17 years)	
	Sleep Ninja	[41]		Aimed to develop a sleep app that offers personalized sleep information, allows flexible completion of sleep diaries, incorporates game-like features, and has an engaging layout. Participants expressed a preference for a trusted developer, offline accessibility, and free access to the app.		N=21 (12-16 years)	
	MIND MORE	[42]		Explored the relationships among subjective sleep quality, memory complaints, and depressive symptoms. Evaluated the usability of MIND MORE among older adults (urban Koreans) and analyzed changes in sleep quality during a 1-week intervention. Participants assessed their adherence to the app.		N=41 (≥60 years)	
	WeChat Mini Program	[43]		Assessed the efficacy of cognitive behavioral therapy in preventing the progression from episodic to persistent insomnia. Supported online self-guided sleep interventions for individuals with persistent insomnia.		N=200 (≥18 years)	
	CBT-I Coach	[44]		Examined the feasibility, acceptability, and effects on adherence and sleep outcomes of using the CBT-I Coach app for cognitive behavioral therapy for insomnia. Patients who utilized the app as intended reported high levels of acceptability.		N=18 (46-50 years)	
	Emohaa	[45]		Evaluated the effectiveness of Emohaa, a cognitive behavioral therapy–based conversational agent, in alleviating symptoms of mental distress. The results indicated significant improvements in mental distress, particularly among individuals experiencing depression and insomnia.		N=301 (20-30 years)	
	XiaoE	[46]		Investigated the impact of XiaoE, a cognitive behavioral therapy–based mental health chatbot, on young adults with depression during the COVID-19 pandemic. The chatbot offered easily accessible and self-directed mental health support.		N=148 (17-34 years)	
	Kokoro-App	[47]		Accessed via the app, the cognitive behavioral therapy program, connected to a remote server, effectively alleviated depression. It gathered information on participant utilization to enhance the program and mobile health features.		N=164 (40 years)	
	Sleep Ninja	[48]		Utilizes cognitive behavioral therapy for insomnia in adolescents through a chatbot format that provides personalized information. It offers psychoeducational content, sleep hygiene tips, and sleep-focused cognitive therapy to improve sleep.		N=47(12-16 years)	
	ExpiWell	[49]		Assessed the feasibility and acceptability of smartphone-based cognitive behavioral therapy interventions for sleep disorders. The study provides a low-intensity, accessible intervention, contributing to the more regular delivery of sleep interventions.		N=14 (22-57 years)	
**Cognitive and emotional support (n=4)**							
	Autonomous AI^a^ chatbot	[50]		Assists individuals with insomnia by utilizing AI and deep learning to enhance interactions and provide cognitive and emotional support. A friendly chatbot was developed to aid in understanding and treating insomnia.		Not mentioned	
	CARO	[51]		Proposes CARO, a chatbot designed to provide empathetic conversations and medical advice for individuals with major depression. It senses context, intent, and emotions to facilitate personalized interactions.		Not mentioned	
	Tess	[52]		Tess is a feasible option for providing emotional support but is not intended to replace trained therapists. Results indicate that AI has the potential to reduce symptoms of depression and anxiety through cognitive behavioral therapy–based conversations.		N=74 (22 years)	
	KANOPEE	[53]		Developed KANOPEE, a smartphone app featuring a virtual agent for autonomous screening and digital behavioral interventions. This study demonstrates the feasibility of using virtual agents and highlights the potential of empathetic human-machine interfaces in eHealth solutions.		N=47 (18-65 years)	
**Examining symptoms or interventions for depression (n=4)**							
	Tess	[54]		Assessed the intervention’s effects on depression and anxiety symptoms in university students using the chatbot Tess. The pilot trial indicated a decrease in anxiety symptoms among the experimental group.		N=181 (18-33 years)	
	DEPRA	[55]		An AI-based chatbot designed for the early detection of depression. Integration with social media attracted over 40 participants, and user surveys indicated high levels of satisfaction.		N>40 (18-33 years)	
	XiaoNan	[56]		Explored the efficacy of chatbot therapy for depression among university students, aiming to provide a convenient, affordable, and interactive self-help psychotherapy approach.		N=83 (19-28 years)	
	DPs	[57]		Proposed a personalized design for conversational agents aimed at treating depression, incorporating transparency and autonomy as key design principles based on interviews with individuals diagnosed with depression.		N=15 (14-17 years)	

^a^AI: artificial intelligence.

### Research Topics

Table 1 shows that the mobile apps discussed in the context of insomnia and depression can be broadly categorized into 3 major areas: cognitive behavioral interventions, cognitive and emotional support, and the examination of symptoms or interventions for depression.

A comprehensive research synthesis revealed that studies focusing on “Cognitive Behavioral Interventions” are particularly prominent, totaling 10 [5,41-49]. This underscores researchers’ concentrated emphasis on cognitive behavioral interventions in mobile apps addressing insomnia and depression. Additionally, 4 studies [50-53] focused on “Cognitive and Emotional Support,” highlighting the importance of mobile apps in providing cognitive and emotional assistance. Furthermore, 4 studies [54-57] were dedicated to “Examining Symptoms or Interventions for Depression,” underscoring a sustained interest in apps aimed at the thorough exploration and management of depressive symptoms.

### Study Participants

The target audience for these apps included young people (aged 13-25 years; n=12) [5,41,43,45,46,48,49,52,54-57] and adults (aged 18 years and older; n=11) [42-47,52-56] (Table 1).

The primary purpose of mobile apps is to provide personalized support [5,50-52,54,56,57]. In addressing stress, depression, and insomnia, these chatbots offer effective tools and resources to help users improve their mental well-being. They deliver tailored advice, CBT exercises [5,41,43,44,46-50,52,56], and emotional support [45,46,53].

This suggests that the design and development of mental health apps should be tailored to the specific needs and characteristics of different age groups to more effectively address mental health challenges across diverse age ranges. By focusing on both young individuals (13-25 years) and adults (18 years and older), these apps aim to provide targeted, relevant, and personalized mental health support and therapy. This approach contributes to innovation and advancement in the mental health field.

### App Functions

The main focus was on early detection, assessment, and screening (n=5) [42,51,54,55,57], counseling and psychological support (n=3) [45,46,53], and CBT (n=11) [5,41,43,44,46-50,52,56]. These apps demonstrate an active exploration of mental health issues, addressing a range of interventions from early detection to personalized psychological support and the provision of CBT.

Primarily adopting CBT-based psychological health chatbots, these apps emphasize feasibility, acceptability, practicality, and safety in their research. They offer diverse solutions that advance the field of mental health technology by prioritizing scientifically grounded approaches. Additionally, they focus on the practical feasibility and acceptability of real-world apps with the potential to achieve comprehensive, profound effects while promoting mental well-being.

### Therapeutic Efficacy

Mobile apps targeting both depression and insomnia have been extensively researched for their therapeutic efficacy. Numerous studies support their positive impact on mental health, demonstrating their effectiveness in addressing both conditions simultaneously. Depression and insomnia often co-occur, exacerbating one another in a vicious cycle. Numerous studies [41-44,46-52,55] have shown that AI-powered chatbots in these apps provide personalized support, including sleep guidance, feedback, and adjustment recommendations, which effectively alleviate insomnia symptoms while positively impacting depression. By offering tools and strategies grounded in CBT, these apps enhance users’ self-regulation skills, helping them cope with negative emotions and stress, thereby reducing the impact of depression.

Furthermore, the emotional support systems integrated into these apps not only promote mental well-being but also empower patients by enhancing their sense of self-efficacy. This enables them to better manage psychological challenges, leading to significant improvements in their quality of life [58]. With technological advancements, mental health apps are showing increasingly diverse and effective therapeutic outcomes, offering more options and support to individuals dealing with both depression and insomnia. For example, the autonomous AI chatbot leverages AI and deep learning to deliver more interactive cognitive and emotional support therapy, with a focus on understanding and treating insomnia. ExpiWell evaluates the feasibility and acceptability of smartphone-based CBT interventions for sleep disorders. MIND MORE explores the relationship between subjective sleep quality and depressive symptoms, assessing its usability among older age Koreans. Emohaa, a CBT-based app, has made significant progress in alleviating symptoms of psychological distress. Woebot examines the feasibility of implementing app-based CBT interventions for adolescents receiving care for depression.

### Mobile App Characteristics

These apps operate across various platforms, including both Android and iOS (n=8) [41-44,48,52-54]. Some are limited to Android only (n=3) [5,46,57], while 1 app supports multiple platforms (n=1) [46]. Additionally, 5 apps did not specify their operating platforms (n=5) [45,47,49-51] (Table 2).

Of these apps, 8 focused on delivering intelligent and personalized mental health support through AI and deep learning techniques. Among the insomnia-related apps, only 2 chatbots, Emohaa and MIND MORE, specifically targeted the connection between depression and insomnia [42,45], with an emphasis on addressing their interrelation. These apps offer effective psychological interventions and guide users in enhancing both sleep quality and mental health. By utilizing AI, deep learning techniques, and personalized processing, they can provide customized advice, techniques, and CBT for addressing depression and insomnia. These chatbots are designed to deliver convenient and accessible support, enabling users to actively engage in and manage their mental health, ultimately leading to positive changes in their daily lives (Table 2).

**Table 2:** Characteristics of chatbots regarding insomnia and depression in the review (n=16).

Targeted area and app name		Description	Main feature	Operating system		Available for download?
**Insomnia**						
	Autonomous AI chatbot	Based on AI^a^ and deep learning, an AI chatbot for people with insomnia that includes natural language processing principles, such as stemming and a bag-of-words. Training data are provided by the app.	The chatbot is designed as a human chat to reduce anxiety through pleasant conversations. The app includes (1) training data, (2) training data “bag of words,” (3) feed-forward neural net, and (4) natural language processing.	Not mentioned		No
	ExpiWell	The ExpiWell app has a prescheduled “My Sleep Programme” and a user-accessible “Resources” section. The content is tailored to participant responses, using branching logic.	The app consists of 6 weekly core modules and a user-initiated “Resources” section. Content with tailored feedback is provided with access to module summaries.	Not mentioned		No
	Sleep Ninja	Sleep Ninja is a CBT^b^ for insomnia–based smartphone app targeting various sleep difficulties in adolescents.	The app uses psychoeducation, stimulus control, sleep hygiene education, and sleep-focused cognitive therapy to improve sleep. Its features are based on user input and sleep profiles. Users complete 6 training sessions and finish with a black belt in sleep.	Android and iOS		Yes
	WeChat Mini Program	eCBTI^c^ will be delivered using the program via the WeChat app. eCBTI is efficient, flexible, and time-saving, and may improve mood and quality of life compared with face-to-face CBTI^d^.	The app provides daily personalized support, encompassing behavioral and cognitive elements, along with relaxation audios and sleep hygiene education following CBTI guidelines.	Android and iOS		Yes
	CBT-I Coach	The CBT-I Coach app was designed to supplement face-to-face CBT^e^ therapy for insomnia and improve adherence to the protocol by completing CBT for insomnia tasks.	The app includes education on sleep processes, positive sleep routines, and environments. It features a sleep diary, tools, guided exercises, reminders for sleep habits, and alarms for prescribed bedtime and wake times.	Android and iOS		Yes
	KANOPEE	KANOPEE is a smartphone app for insomnia; it uses a virtual agent for autonomous screening and interventions during COVID-19.	KANOPEE uses C# in Unity 3D with a virtual agent named Louise, whose natural voice and body movements were recorded using motion capture technologies. The interaction scenario adapts to the user’s answers through decision trees.	Android and iOS		Yes
**Depression**						
	XiaoNan	XiaoNan is a chatbot powered by open-source conversational AI RASA. Its text content is based on CBT principles approved by professional therapists.	XiaoNan accepts both text and voice messages. Voice messages are transcribed using a natural speech recognition service from the IFLYTEK open platform before being processed by the chatbot’s natural language understanding module.	Windows, macOS, Android, and iOS		Yes
	Woebot	Woebot uses a fully automated, relational conversational agent to deliver evidence-based therapeutic elements in brief conversations.	This self-guided intervention uses natural language processing and machine learning to incorporate elements of CBT, interpersonal psychotherapy for adolescents, and dialectical behavior therapy.	Android		Yes
	XiaoE	XiaoE is a CBT-based chatbot, which interacts automatically with users through text, images, and voice, primarily for depression screening, prevention, and self-help.	Its chatbot dialog system is built on the open-source RASA framework. Utilizes natural language processing and deep learning techniques.	Android		Yes
	DPs	The chatbot prototype explores personalized agents with various elements and degrees.	The system allows users to customize their name, gender, avatar, and social roles. It supports personalized modules for behavioral activation and sleep hygiene, with content selected based on user feedback.	Android		Yes
	Tess	Tess is an AI chatbot for depression and anxiety. It supports therapy-based conversations and adjusts interventions based on user feedback.	Tess is an AI chatbot that supports CBT, emotion-focused therapy, and solution-focused brief therapy.	Android and iOS		Yes
	DEPRA	The DEPRA chatbot supports early detection of depression.	DEPRA, developed using Dialogflow and Node.js, communicates with participants via Facebook Messenger. Data are stored in an AWS^f^ database managed with a MySQL Workbench client.	Android and iOS		Yes
	CARO	CARO is an empathetic chatbot app that provides medical advice for major depression.	This study used automated metrics, namely, the Bilingual Evaluation Understudy score and Bidirectional Encoder Representations from Transformers score, and the text is classified into conversations or medical advice.	Not mentioned		No
	Kokoro-App	A smartphone CBT app that supports self-monitoring, behavioral activation, and cognitive restructuring skills.	The app has 4 parts: sessions, mind maps, actions, and thoughts. It allows users to enter details of situations, emotions, thoughts, and behaviors.	Not mentioned		No
**Insomnia and depression**						
	Emohaa	A CBT-based conversational agent for reducing mental distress that has demonstrated significant improvements in depression and insomnia.	This conversational agent has 2 platforms: a template-based platform with predefined options and exercises based on CBT principles and a generative dialog platform that allows open-ended conversations and provides emotional support.	Not mentioned		No
	MIND MORE	The app analyzed the relationships among sleep quality, memory complaints, depressive symptoms, and app usability and adherence. It is a multimodal CBT for insomnia app with sleep education components.	The main sleep hygiene education program has 3 sessions that include quizzes to evaluate user learning. It helps users understand the mechanisms sustaining insomnia and correct unhelpful sleep-related beliefs, anxiety, arousal, and sleep-disruptive habits. This improves adherence to the app’s recommendations.	Android and iOS		Yes

^a^AI: artificial intelligence.

^b^CBT: cognitive behavioral therapy.

^c^eCBTI: electronic cognitive behavioral therapy for insomnia.

^d^CBTI: cognitive behavioral therapy for insomnia.

^e^CBT: cognitive behavioral therapy.

^f^AWS: Amazon Web Services.

### The Feasibility and Acceptability of Mobile Apps in Sleep and Depression Interventions

Taylor et al [49] highlighted the feasibility, acceptability, and potential usefulness of smartphone-based CBT interventions delivered through apps aimed at treating sleep problems in individuals with psychosis, demonstrating that CBT is both feasible and acceptable.

In another study, Nicol et al [5] examined the feasibility of providing an app-based CBT intervention for adolescents aged 13-17 years with depression. The study assessed the feasibility, acceptability, practicality, and safety of using a CBT-based chatbot over a 12-week trial. The results indicated that the use of the CBT-based chatbot was feasible, acceptable, and safe for adolescents experiencing moderate depressive symptoms.

As sleep intervention tools, mobile apps not only help improve sleep but also record users’ sleep data, provide sleep tracking and reporting, and enhance users’ understanding of their sleep habits and progress [59]. Mobile apps have demonstrated the potential to improve sleep disturbances and offer more convenient and personalized support [7,28]. Similarly, these apps have shown strengths in depression interventions by providing emotional support, which can, in turn, help alleviate depression symptoms [60].

### The Effectiveness of Mobile Apps as a Depression and Sleep Intervention Tool

Two studies [42,45] evaluated the effectiveness of mobile apps as alternative intervention tools for depression and sleep. Sabour and colleagues [45] confirmed that the demand for mental health support is increasing. Their study included 134 participants, and the results indicated that those using Emohaa experienced significant improvements in symptoms of mental distress. The inclusion of emotional support had a complementary effect on both depression and insomnia. Participants expressed high satisfaction with the Emohaa platform, viewing it as a practical and effective tool. These results offer additional options for individuals seeking emotional support.

Chung et al [42] examined the relationship between sleep quality, depressive symptoms, and the usability of a smartphone-based self-help CBT for an insomnia app called MIND MORE for older adults. They compared participants’ sleep quality before and after a 1-week intervention period, along with an assessment of adherence to the app. During the study, 41 participants used the app and completed usability questionnaires both before and after the intervention. The results indicated significant improvements associated with the use of the MIND MORE app.

### AI Chatbots’ Potential and Applications

These apps showcase the diverse capabilities of AI in chatbots, incorporating technologies such as natural language processing (NLP), ideographic recognition, machine learning, and sentiment analysis [13,61]. These advancements hold the potential for chatbots to be utilized in health care, enabling efficient, equitable, and personalized care. They can assist clinicians in understanding patient conditions and provide decision aids [15,62,63]. Applied functional technologies for AI chatbots encompass a range of components and features that inform future designs and specific apps. Their features are as follows.

An autonomous AI chatbot incorporates various components, including training data, a bag-of-words, a feed-forward neural network, and NLP. These components enable effective understanding and response to user inputs [50].

Emohaa is a generative dialog platform designed to facilitate open-ended conversations and provide emotional support. Users can engage in discussions with a chatbot, allowing them to freely express their thoughts and feelings [45].

XiaoNan is a natural speech-recognition service that utilizes the “IFLYTEK Open Platform” to convert spoken language into text. The chatbot’s natural language understanding module processes this text to effectively comprehend user queries [56].

Woebot utilizes both NLP and machine learning techniques, incorporating elements of CBT, interpersonal psychotherapy for adolescents, and dialectical behavior therapy. By personalizing conversations, Woebot aids adolescents in developing emotion regulation skills for their daily lives [5].

XiaoE is a dialog system built on the open-source RASA framework. By utilizing NLP and deep learning techniques, it provides personalized modules for behavioral activation and sleep hygiene, enabling users to improve their daily routines and sleep patterns [46].

Tess provides therapy-based conversations developed by mental health experts, offering individuals professional support and guidance to manage their mental health [52].

DEPRA utilizes Dialogflow and Node.js to communicate with participants via Facebook Messenger. By leveraging Facebook’s AI Empathetic Dialogue and a web-scraped Medical Q&A data set, the data are stored in an AWS database managed by a MySQL Workbench client. AWS Educate is used to save all interaction records [55].

CARO utilizes Facebook’s AI Empathetic Dialogue and a web-scraped Medical Q&A data set. It uses automated metrics, such as the Bilingual Evaluation Understudy and Bidirectional Encoder Representations from Transformers scores, to assess text, which is classified into conversational or medical advice. Two long short-term memory–based models were trained to generate empathetic responses and provide medical answers [51].

## Discussion

### Principal Findings

We reviewed articles published between 2017 and 2023 and found that mobile apps related to insomnia and depression primarily focus on cognitive behavioral interventions. Additionally, the review revealed 8 apps that emphasize the use of AI and deep learning technologies to provide intelligent and personalized mental health support. Directions for future research are discussed in the following sections.

### AI and Deep Learning for Personalized Mental Health Support With Intelligence

This systematic review investigated mobile apps, categorized the chatbot-themed features of insomnia and depression apps, and explored the correlation between insomnia and depression. It aims to provide comprehensive insights into the common features and therapeutic approaches of chatbot apps, thereby contributing to the improvement of sleep and mental health.

A total of 9 reviewed apps focused on utilizing AI and deep learning technologies to provide intelligent and personalized mental health support [5,45,46,49-51,54-56]. These apps emphasized early detection, assessment, screening, counseling, psychological support, and CBT. CBT-based chatbots record users’ sleep data, provide sleep tracking and reporting, and enable users to better understand their sleep habits and progress. They also offer effective psychological interventions and guidance to improve sleep quality and mental health, along with customized advice and techniques. The goal is to make mental health support readily available and convenient for users. By proactively engaging in and managing their mental health, individuals can achieve positive changes and enhance their daily lives.

Specifically, an autonomous AI chatbot integrates various components, including training data, bag-of-words training data, feedforward neural networks, and NLP, to effectively understand and respond to user input [50]. For example, Emohaa is a generative dialog platform that facilitates open-ended conversations and offers emotional support, enabling users to freely express their thoughts and emotions [45]. XiaoNan, part of the “IFLYTEK Open Platform,” converts spoken language into text, which is then processed by the chatbot’s NLP module to comprehend user queries [56]. Woebot combines NLP and machine learning techniques to integrate CBT, interpersonal psychotherapy for adolescents, and dialectical behavior therapy, helping adolescents develop emotion-regulation skills through customized conversations [5]. XiaoE, built using RASA, utilizes NLP and deep learning to create personalized behavioral activation and sleep hygiene modules aimed at improving users’ routines and sleep patterns [46]. Tess offers therapy-based conversations to provide professional mental health support and guidance [52]. DEPRA uses Dialogflow and Node.js, leveraging Facebook AI Empathetic Dialogue and a web-scraped Medical Q&A data set, which is stored in an AWS database managed by MySQL Workbench. AWS Educate is used to save the interaction records [55]. CARO uses Facebook AI Empathetic Dialogue along with a web-scraped Medical Q&A data set to evaluate text using Bilingual Evaluation Understudy and Bidirectional Encoder Representations from Transformers scores [51]. It provides empathetic responses and medical advice through 2 long short-term memory–based models. The diversity of these chatbots enriches the choices available within apps, catering to the varied needs of different users.

### Mobile Apps Offer the Potential to Improve Insomnia in Depression Support

Mobile apps hold significant potential for addressing insomnia and depression. The primary focus has been on early detection, assessment, and screening (n=5) [42,51,54,55,57]; counseling and psychological support (n=3) [45,46,53]; and CBT (n=11) [5,41,43,44,46-50,52,56]. These apps represent a proactive exploration of mental health issues, providing personalized psychological support and CBT from the early detection stage. This comprehensive approach addresses the issues of depression and insomnia by offering users multilayered support. This includes screening and assessment features, real-time counseling, psychological support, and therapy grounded in CBT.

The focal point of these studies has been the diversity of apps within the mental health field, encompassing everything from early intervention to specific therapeutic methods. By leveraging AI and deep learning technologies, these apps can comprehensively understand users’ needs and provide tailored solutions. The application of CBT is particularly noteworthy, reflecting the ongoing exploration of therapeutic methods and the pursuit of innovative technological apps.

Overall, these apps have demonstrated significant potential to improve mental health by offering users convenient and personalized support. However, as this field continues to evolve, several directions for future research warrant further exploration. Enhancing the effectiveness of these apps, improving the accuracy of screening and assessment, and more effectively integrating psychological support with traditional therapeutic approaches are all valuable avenues for future investigation.

### Limitations

This review investigates chatbot apps within mental health–related mobile apps, focusing on the use of AI and deep learning technologies. During the screening process, researchers examined various aspects, including research topics, participant characteristics, app features, and therapeutic outcomes, resulting in the inclusion of 18 articles. However, this review also has certain limitations. First, subjectivity in the screening process may have been influenced by the researchers’ personal judgments. Second, the inclusion of only 18 articles raises questions about whether this selection adequately represents the diversity and comprehensiveness of the entire field, as some relevant studies might have been overlooked. Additionally, it is worth noting that research on the relationship between depression and sleep is relatively scarce, indicating that this area presents a promising opportunity for further investigation. We hope our review serves as a foundation for research in this field and stimulates further academic discussion. While AI and deep learning are evolving rapidly, the long-term effects of their apps remain unclear, warranting additional research. This review offers a preliminary understanding of the use of chatbots in mental health–related mobile apps. However, readers should exercise caution in considering these potential limitations.

This study identified several risks associated with mobile apps targeting insomnia and depression, including the lack of well-defined clinical designs and usage guidelines, insufficient privacy protection, inadequate monitoring to prevent harm from inappropriate user interactions, and challenges in integrating these apps with existing medical models. These issues can result in misinterpretation and potentially ineffective or harmful interventions. In addition, chatbots may lack crisis warning mechanisms and appropriate privacy safeguards [64,65]. Although mobile apps targeting insomnia and depression have shown promise in providing suitable treatment, there is a need for stronger and more robust evidence to comprehensively support their effectiveness and acceptability. Further research is necessary to address these issues and enhance the efficacy of insomnia-related interventions within mobile apps for mental health. In particular, integrating chatbots into existing health care models faces challenges due to potential misconceptions, which could lead to ineffective or harmful interventions. While advancements have been made in the field of mental health, additional high-quality research is necessary to confirm the effectiveness and acceptability of mobile apps in this area. Further studies will help address these challenges and enhance the effectiveness and reliability of mobile apps targeting depression and insomnia symptoms.

### Future Prospects

According to our study, agent prompts that integrate human intelligence with deep learning offer intelligent and personalized cognitive and emotional support. This creates numerous opportunities for researchers to combine NLP with human intelligence in developing chatbots specifically designed to address mental health issues. These mental health chatbots can serve as effective sleep intervention tools by providing assistance, recording users’ sleep data, and offering sleep tracking and reporting capabilities, thereby enabling individuals with depression to gain a better understanding of their sleep habits and progress. When combined with recommendations from experienced therapists to establish guidelines, these chatbots can be more effective in alleviating the severity of insomnia associated with depression, particularly because long-term interventions have proven to be more effective in improving insomnia severity.

### Conclusions

After synthesizing critical information from previous literature on research topics, participants, app functionalities, therapeutic effects, operating platforms, technical foundations, and functional features, as well as feasibility and acceptability, this review offers a systematic summary of the application and effectiveness of mobile apps in the field of mental health. This will serve as a guiding framework for related studies and future developments. Furthermore, by analyzing app technologies—particularly, the use of AI and deep learning techniques to provide personalized mental health support—mobile apps have shown significant potential in addressing insomnia in individuals with depression. Our research outlines specific directions for future exploration in this domain and contributes to the existing literature.

Currently, AI is unable to fully replace mental health treatment, primarily due to its limitations in replicating the comprehensive skill set of psychiatrists and psychotherapists. Although AI can utilize patient information for diagnosis and prognostic predictions, integrating the diverse layers of biological, psychological, and social data needed to formulate a holistic treatment plan remains a challenge. AI struggles to perceive nuanced emotions, exhibit empathic responses, interpret relational dynamics, and observe subtle behavioral and emotional changes. While AI cannot fully replace existing mental health treatments, integrating AI technology with human therapeutic approaches holds the potential to enhance treatment outcomes, foster innovation, and drive advancements in mental health.

## Appendix

Multimedia Appendix 1 PRISMA (Preferred Reporting Items for Systematic Reviews and Meta-Analyses) 2020 checklist.

Multimedia Appendix 2 Methodological protocol amendments and clarifications log.

Multimedia Appendix 3 Indexed and keyword terms.

## Abbreviations

AI: artificial intelligence
CBT: cognitive behavioral therapy
NLP: natural language processing
PRISMA: Preferred Reporting Items for Systematic Reviews and Meta-Analyses
