# Supplementary material for: Exploring the Role of Mobile Apps for Insomnia in Depression: Systematic Review
Source: J Med Internet Res. 2024 Oct 18;26:e51110. doi: 10.2196/51110 (PMC11530740; doi:10.2196/51110)
Supplement: Multimedia Appendix 2 [file jmir_v26i1e51110_app2.docx]

## Multimedia Appendix 2

**Methodological protocol amendments and clarifications log.**

| **Stage/Date** | | **Change or Clarification** |
| --- | --- | --- |
| **Literature search** | | |
|  | 03/15/23 | - Conducted a comprehensive systematic search across databases including PubMed, SCOPUS, and ISI Web of Science. - Search strings included "mobile applications," "chatbots," "insomnia," "insomnia treatment," "depression," "internet," "smartphones," and "mobile health." |
|  | 03/18/23 | - The search string "(internet, smartphones, and mobile health)" has been removed. |
| **Eligibility Screening** | | |
|  | 05/01/23-06/30/23 | - ***Criteria for Inclusion:*** 1. Studies must focus on mobile applications addressing   both depression and insomnia. 2. Participants must include young people or adults. 3. Studies must provide data on treatment efficacy. - ***Criteria for Exclusion:*** 1. Studies that do not focus on both depression and   insomnia. 2. Studies that do not involve mobile applications in the   context of depression or insomnia. 3. Articles not published in peer-reviewed journals or   lacking credibility in methodology. - ***Screening Process: A. Overview of Screening Process:*** 1. Initial screening based on titles and abstracts to   identify potentially relevant studies. 2. Full-text review of selected studies to confirm   eligibility based on inclusion and exclusion criteria. ***B. Initial Screening (Titles and Abstracts):*** 1. Exclude studies that clearly do not meet the inclusion criteria (e.g., unrelated topics, different interventions). ***C. Full-Text Review:*** 1. Retrieve full texts of articles passing initial screening. 2. Assess full texts against detailed eligibility criteria to   ensure relevance and methodological rigor. ***D. Independent Review and Verification:*** 1. Two reviewers independently conduct title and abstract screening, as well as full-text screening, to ensure consistency and objectivity. 2. Once eligible studies are identified, data extraction is   performed. |
| **Data Extraction** | | |
|  | 07/16/23-08/05/23 | - ***A. Content Extraction:*** 1. Extracted data includes study design, participant   characteristics, intervention measures, primary   outcomes, and key findings. 2. Special focus on the functionalities of mobile   applications, effectiveness assessment, technological   applications, and specific contents for managing   depression and insomnia. - ***B. Review and Confirmation:*** 1. Data extraction is conducted by one reviewer and   verified by a second reviewer to ensure accuracy and   completeness. |
| **Data Analysis** | | |
|  | 09/01/23-10/28/23 | - ***A. Analysis Methods:*** 1. Statistical or qualitative analysis is employed to   examine the effects and impacts of mobile applications   on managing depression and insomnia. 2. Focus on comparing different types of mobile   applications, evaluating the effectiveness of various   interventions, and assessing the influence of   technological applications. |
|  |  | - ***B. Findings and Discussion:*** 1. Analyze the application of mobile applications in early   detection, assessment, psychological counseling, and   cognitive behavioral therapy (CBT). 2. Discuss the feasibility, acceptability, and potential   efficacy of mobile applications compared to traditional   face-to-face therapies in enhancing sleep and mental   health outcomes. |
