# Supplementary material for: Exploring the Role of Mobile Apps for Insomnia in Depression: Systematic Review
Source: J Med Internet Res. 2024 Oct 18;26:e51110. doi: 10.2196/51110 (PMC11530740; doi:10.2196/51110)
Supplement: Multimedia Appendix 3 [file jmir_v26i1e51110_app3.docx]

## **Multimedia Appendix 3**

| Database | Indexed and keyword terms |
| --- | --- |
| PubMed | ((depression [Mesh] OR depression OR depressive) AND (insomnia [Mesh] OR insomnia OR "sleep disorder" OR "sleep disturbance" OR "insomnia treatment") AND ("mobile applications"[Mesh] OR "mobile applications" OR chatbots [Mesh] OR chatbots)) |
| Scopus | TITLE-ABS-KEY ((depression OR depressive) AND (insomnia OR "sleep disorder" OR "sleep disturbance" OR "insomnia treatment") AND ("mobile applications" OR chatbots)) |
| Web of Science | TOPIC: ((depression OR depressive) AND (insomnia OR "sleep disorder" OR "sleep disturbance" OR "insomnia treatment") AND ("mobile applications" OR chatbots))  Indexes=SCI-EXPANDED, SSCI, A&HCI, CPCI-S, CPCI-SSH, ESCI  Timespan=2017-2023 |
